# Influence of Gender on Associations of Obstructive Sleep Apnea Symptoms with Chronic Conditions and Quality of Life

**DOI:** 10.3390/ijerph15050930

**Published:** 2018-05-07

**Authors:** Sarah Appleton, Tiffany Gill, Anne Taylor, Douglas McEvoy, Zumin Shi, Catherine Hill, Amy Reynolds, Robert Adams

**Affiliations:** 1 Discipline of Medicine, University of Adelaide, The Queen Elizabeth Hospital Campus, Woodville 5011 SA, Australia; robert.adams@adelaide.edu.au; 2 Adelaide Medical School, University of Adelaide, Adelaide 5005 SA, Australia; tiffany.gill@adelaide.edu.au (T.G.); anne.taylor@adelaide.edu.au (A.T.); zumin.shi@adelaide.edu.au (Z.S.); 3 Adelaide Institute for Sleep Health, A Flinders Centre of Excellence, College of Medicine & Public Health, Flinders University, Bedford Park 5042 SA, Australia; doug.mcevoy@flinders.edu.au; 4 Rheumatology Unit, The Queen Elizabeth Hospital, Woodville 5011 SA, Australia; Catherine.Hill@sa.gov.au; 5 The Appleton Institute, CQUniversity Australia, 44 Greenhill Rd, Wayville SA 5034, Australia; a.reynolds@cqu.edu.au

**Keywords:** obstructive sleep apnea, epidemiology, gender, health status, quality of life

## Abstract

Women are less likely than men to be diagnosed with obstructive sleep apnea (OSA). We examined contemporary gender differences in symptoms, health status, and quality of life associated with diagnosed OSA and OSA symptoms in a population-based sample. A 2015 postal/on-line questionnaire of 2889 active participants of The North West Adelaide Health Study (response rate = 54%, male n = 704; female n = 856; age 30–100 years) assessed previously diagnosed OSA, OSA symptoms, insomnia, doctor-diagnosed medical conditions, and the SF-36. In weighted analyses, self-reported diagnosed OSA (men: 12.6%, n = 95; women: 3.3%, n = 27) and OSA symptoms (men: 17.1%; women: 9.7%) were more common in men. Diagnosed OSA showed stronger adjusted associations with typical OSA features in women, including obesity (women-odds ratio (OR), 95% CI: 5.7, 1.9–17.1, men: 2.2, 1.2–4.0), daytime sleepiness (women: 6.4, 2.7–15.6, men: 3.3, 2.1–5.4), and loud snoring (women: 25.4, 9.4–69.1, men: 8.7, 5.2–14.4). Diagnosed OSA was independently associated with cardiovascular disease (CVD) in men, and in women with high cholesterol, respiratory disease, insomnia, and reduced SF-36 Physical Component Summary score. In both sexes, OSA symptoms were significantly associated with depression, insomnia, and moderate to severe impairments in SF-36 physical and mental component summary scores. Diagnosed women showed clinical characteristics overtly related to OSA. A higher index of clinical suspicion of OSA may be required in women for a condition regarded as male-predominant to increase equity in health outcomes.

## 1. Introduction

Obstructive sleep apnea (OSA) is a disorder characterized by snoring and repetitive upper airway collapse during sleep that can be complete (apnea) or partial (hypopnea). This leads to repetitive acute episodic oxygen desaturation, fragmentation of sleep, and marked negative intrathoracic pressures that negatively impact on cardiovascular and metabolic health through adverse effects on endothelial function, increased blood pressure and sympathetic nervous system activation, and inflammation.

The importance of OSA in women has received increased recognition in the past two decades and contemporary population-based studies report a prevalence of objectively determined moderate to severe OSA in women of 20–23% [1,2]. Population-based studies consistently show objectively determined OSA is twice as common in men compared to women [2], which may be accounted for by sex differences in airway pathophysiology [3,4,5,6]. However, women with OSA symptoms are less likely than men to be diagnosed and treated for OSA [7], and women are under-represented in studies of clinic samples [6]. Population-based [8] but not all clinic-based samples [9,10,11,12] suggest that typical OSA symptoms (snoring and daytime sleepiness) are similar in both genders. However, clinic-based samples have identified a significantly increased propensity for women with OSA to report depression [13], insomnia, restless legs, and morning headaches, to be older and more obese compared to men [9,10,11,12,14], and demonstrate a higher rapid eye movement (REM) sleep apnea hypopnea index [14,15]. Women may also have greater impairments in quality of life and healthcare utilisation related to OSA than men [16,17].

Awareness of the importance of OSA in Australia has been high for several decades with the first report of an effective treatment in 1981 by Sullivan et al. [18] and the relatively open access to sleep studies. Universal health insurance and reimbursement for sleep studies has led to a 240% increase in the provision of government-funded sleep apnea diagnostic services in Australia since 2005. However, the dramatic increase in provision of sleep studies does not guarantee that the right men or women are being screened and identified for treatment. We have previously identified a high prevalence of objectively measured undiagnosed OSA in community-dwelling men aged over 40 years [19]. We hypothesize that there is also a large burden of women with symptoms of sleep-disordered breathing without a diagnosis of OSA in the community.

The aim of this study was to obtain a contemporary picture of the gender differences in polysomnography-diagnosed OSA and symptoms suggestive of undiagnosed OSA in a population-based study of community-dwelling adults. We sought to determine the characteristics of men and women with diagnosed OSA and possible undiagnosed OSA and their associations with chronic conditions and quality of life in The North West Adelaide Health Study cohort.

## 2. Materials and Methods

Data presented here were collected in the 2015 postal follow-up of the 2889 remaining active participants of The North West Adelaide Health Study (NWAHS), of whom 1560 returned questionnaires (response rate = 54%) and were aged between 30 years and 100 years. The NWAHS is an ongoing biomedical cohort study of 5850 randomly selected adults aged at least 18 years at the time of recruitment in 1999/2003 from the north-west region of Adelaide, South Australia, of whom 4060 participants underwent clinic assessment [20]. Subsequent follow-up clinic assessments occurred in 2004/2006 (Stage 2 n = 3206) and 2008/2010 (Stage 3 n = 2487). The Human Research Ethics Committees of the Central Adelaide Local Health Network approved the conduct of the postal survey (HREC/15/TQEH/127) and consent was implicitly granted by the participants’ completion of the survey.

### 2.1. Sleep Variables

Participants answered questions regarding a previous diagnosis of OSA (Have you been diagnosed with sleep apnea with an overnight sleep study?), OSA symptoms (Has your snoring ever bothered other people, or is it louder than talking or loud enough to be heard through closed doors? Has anyone noticed that you stop breathing during your sleep?), and nocturia. Excessive daytime sleepiness was determined by a yes response to two or more of the following three questions (Do you often feel tired, fatigued or sleepy during daytime? Do you feel sleepy when sitting quietly during the day or early evening? During the past month, how often have you had trouble staying awake while driving, eating meals, or engaging in social activity? The Insomnia Severity Index was administered with a score of ≥10 considered insomnia in epidemiological studies [21]. We classified those with symptoms of sleep-disordered breathing (loud snoring and daytime sleepiness) separately as an OSA symptom group to distinguish them from the “no OSA diagnosis” group. We then compared the sleep health, general health, and quality of life characteristics of these groups (diagnosed OSA, OSA symptoms, no OSA) by gender and socio-demographics.

### 2.2. Chronic Conditions

Self-reported doctor-diagnosed conditions were assessed, including diabetes (excluding gestational diabetes); cardiovascular disease/stroke (CVD/stroke: at least one of heart attack; stroke; angina; transient ischemic attack/mini-stroke; kidney/renal disease; high blood pressure; high cholesterol; asthma; and smoking-related lung condition (e.g., chronic obstructive pulmonary disease (COPD)/chronic obstructive airways disease (COAD)). Diabetes, hypertension, and hormone replacement therapy (HRT) were also determined from prescribed medication use data. Smoking status was assessed and body mass index (BMI, kg/m^2^) was calculated from self-reported height and weight. Depression was determined by a score of ≥16 on the Center for Epidemiologic Studies Depression Scale (CESD). Obstructive lung disease was classified based on self-report of asthma or COPD (2015) and/or spirometric assessment that occurred in 2008–2010. COPD was classified as post-bronchodilator forced expiratory volume in 1 s (FEV_1_)/forced vital capacity (FVC) <0.7 and asthma classified as post-bronchodilator FEV_1_ reversibility (at least 12% and 200 mL) or self-reported doctor-diagnosed current asthma as previously described [22]. The postal questionnaire also included the SF-36 quality of life questionnaire and demographic items.

### 2.3. Statistical Analysis

Data were analyzed using SPSS version 24.0 (IBM Corporation, Armonk, NY, USA). Pearson Chi^2^ tests determined significant differences in distribution of demographics, sleep variables, and comorbidities by sex and OSA status. Logistic regression models adjusted for age and BMI determined the association of OSA status with chronic conditions (dependent variable). Logistic regression models for respiratory disease were additionally adjusted for smoking status. *t*-Tests and ANOVA determined mean age, BMI, and SF-36 physical component summary (PCS) and mental component summary (MCS) scores in relation to sex and OSA status. Multiple linear regression models were conducted with OSA status dichotomized (diagnosed versus no OSA, OSA symptoms versus no OSA) as primary independent variables to predict PCS and MCS scores with adjustment for age and BMI. PCS and MCS scales are scored to generate a mean of 50 with an SD of 10 [23]. Where applicable, all analyses were weighted to the population of the northern and western suburbs of Adelaide. In Stage 1 of the NWAHS, data were weighted by region (western and northern health regions), age group, sex, and probability of selection in the household to the Australian Bureau of Statistics 1999 Estimated Resident Population and the 2001 Census data to reflect the population of interest. Postal survey data were reweighted using 2011 Census data whilst retaining the original weight from Stage 1 in the calculation. We have previously shown that weighting of data may overcome the inadequate representativeness of the respondents of follow-up study waves [24].

## 3. Results

Demographic and sleep characteristics of 1560 participants overall and by sex are shown in Table 1. While there was no significant difference in mean BMI, men were less likely to be of healthy weight (BMI < 25) and were more likely to be overweight compared to women. Women were significantly more likely than men to have lower levels of educational attainment and household income and less likely to be cohabitating with a partner. The weighted prevalence of self-reported diagnosed OSA in men was 12.6% (n = 95) compared to 3.3% (n = 27) in women. Men were significantly more likely to report OSA symptoms (loud snoring, witnessed apneas), but were less likely to have insomnia compared to women.

### 3.1. Diagnosed OSA

#### 3.1.1. Sleep Characteristics and OSA Risk Factors

Both men and women with diagnosed OSA were significantly more likely to report loud snoring and witnessed apneas and increased adiposity than those with no OSA diagnosis (Table 2). In those with diagnosed OSA, compared to men, women were not significantly less likely to report snoring (odds ratio (OR), 95% CI: 0.9, 0.4–2.0) or breathing pauses (1.6, 0.6–4.0).

Over three quarters of women with diagnosed OSA had a BMI ≥30.0 kg/m^2^ (mean = 34.7 kg/m^2^) compared to half of men (mean = 30.8 kg/m^2^). Diagnosed OSA showed no association with facilitating factors for diagnosis, including increased private health insurance ownership or having a spouse. Compared to participants without OSA, diagnosed OSA showed stronger adjusted associations with typical OSA features in women compared to men, including obesity (men-odds ratio (OR), 95% CI: 2.2, 1.2–4.0; women: 5.7, 1.9–17.1), reporting two of three sleepiness problems (men: 3.3, 2.1–5.4; women: 6.4, 2.7–15.6), and loud snoring (men: 8.7, 5.2–14.4; women: 25.4, 9.4–69.1).

An association with age ≥65 years (1.7, 1.1–2.6) and current smoking (age-adjusted OR, 95% CI: 2.1, 1.1–4.0) were seen in men only while an age-adjusted association with insufficient physical activity was present in women only (2.1, 1.1–4.2). A relationship of diagnosed OSA with nocturia in men did not persist after adjustment for age and BMI.

#### 3.1.2. Chronic Conditions

**Figure 1:**
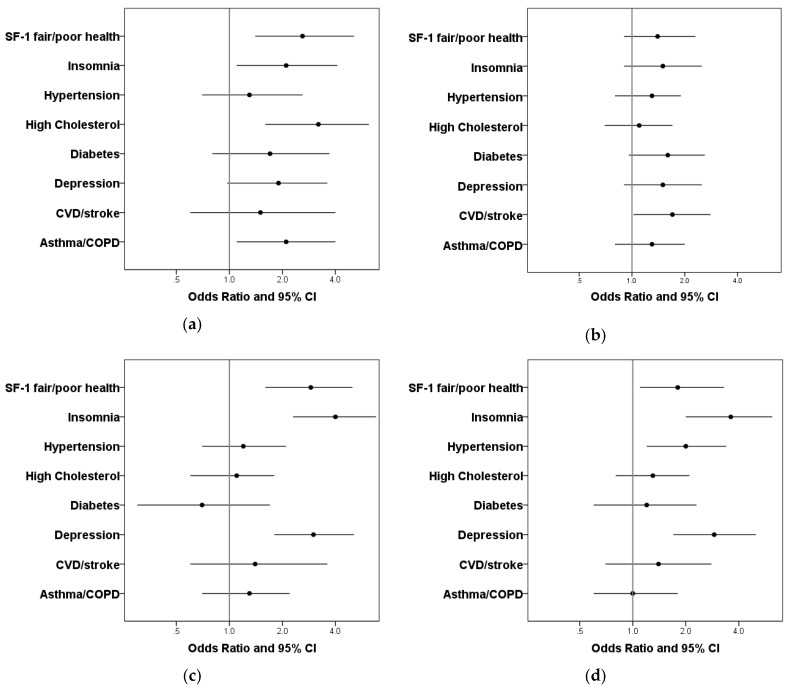
Adjusted odds ratio (OR) and 95% confidence intervals (CI) for comorbid conditions associated with diagnosed OSA and OSA symptoms not diagnosed (**a**) Women-diagnosed OSA; (**b**) Men-diagnosed OSA; (**c**) Women-OSA symptoms; (**d**) Men-OSA symptoms; adjusted for age and body mass index (BMI); models for lung disease, cardiovascular disease, hypertension were adjusted for age, BMI, and smoking. Depression: Center for Epidemiologic Studies Depression Scale score ≥16; SF-1: Question one of the SF-36: In general, would you say your health is? (Excellent; very good; good; fair; poor). CVD = cardiovascular disease; COPD = chronic obstructive pulmonary disease.

In men, diagnosed OSA was significantly associated with cardiometabolic conditions, kidney disease, and self-reported fair to poor general health (Table 3). However, after adjustment for age and BMI, positive associations of diagnosed OSA persisted only for CVD (*p* < 0.05, Figure 1). In contrast, women with diagnosed OSA were significantly more likely to report health conditions, including diabetes, high cholesterol, lung disease, fair to poor general health, and insomnia (Table 2), and except for diabetes, these associations persisted after adjustment for age and BMI (Figure 1). An adjusted association with depression was positive but did not achieve statistical significance (*p* = 0.06).

#### 3.1.3. Health-Related Quality of Life

In both men and women with diagnosed OSA, unadjusted mean (SD) SF-36 physical component summary (PCS) and mental component summary (MCS) scores were significantly impaired compared to those with no OSA (Table 4) and did not differ significantly from those in the OSA-symptoms-only group. After adjustment for age and BMI in linear regression analyses, diagnosed OSA in men was not associated with significant impairments in PCS or MCS scores. In women with diagnosed OSA, significant reductions in PCS scores were observed.

### 3.2. OSA Symptoms Not Diagnosed

#### 3.2.1. Sleep Characteristics and OSA Risk Factors

The prevalence of OSA symptoms (defined as loud snoring with two of three daytime sleepiness problems) was 17.1% in men and 9.7% in women. Witnessed apneas and increased adiposity were significantly more common in men and women with OSA symptoms compared to those classified as no OSA (Table 2). In those with OSA symptoms, men were significantly more likely than women to report breathing pauses (age- and BMI-adjusted OR, 95% CI = 3.6, 1.4–9.1).

OSA symptoms were significantly associated with younger age and insufficient physical activity (age-adjusted OR, 95% CI: 1.8, 1.1–3.2) in women, and an association with current smoking was seen in men (2.9, 1.4–5.7). Significant associations of nocturia with OSA symptoms were seen in men and women, which persisted only in men after adjustment (men: 2.3, 1.4–3.8; women: 1.4, 0.8–2.4).

#### 3.2.2. Chronic Conditions

In both men and women, OSA symptoms were significantly associated with depression, fair to poor general health (Table 3), and insomnia (Table 2). In men, OSA symptoms were also significantly associated with hypertension and high cholesterol. All of these associations (with the exception of high cholesterol in men) persisted after adjustment for age and BMI and smoking where relevant (Figure 1).

#### 3.2.3. Health-Related Quality of Life

Unadjusted mean (SD) SF-36 physical component summary (PCS) and mental component summary (MCS) scores were significantly reduced in both men and women with OSA symptoms compared to those with no OSA (Table 4), and these severe impairments in scores, equating to half a standard deviation or more, persisted after age and BMI adjustment.

## 4. Discussion

In a population-based sample of adults aged at least 30 years, men were over 3 times more likely to report having been diagnosed with OSA than women. This differential is larger than the twofold difference found in population studies where the diagnosis was made in unselected participants with overnight sleep studies, raising the possibility of under-diagnosis in women in our sample. Loud snoring, obesity, and sleepiness were associated with diagnosed OSA in both men and women; however, women had a higher level of obesity. Sex-specific differences in the associations of diagnosed OSA with chronic conditions were seen.

Consistent with recent results in a large Turkish clinical cohort [11], our findings suggest that a diagnosis of OSA may rely upon presentation features that differ depending on sex. Women who report being diagnosed with OSA are more likely than diagnosed men to have the classical clinical features of the syndrome (obesity, loud snoring, daytime sleepiness) as well as have independent associations with insomnia, respiratory disease, high cholesterol, and poor overall health. In contrast, men may be referred for investigation and diagnosed with OSA as part of CVD secondary prevention. These findings may reflect different elements at work in the investigative and diagnostic process. Prior population-based studies indicate that women with OSA are more likely to suffer insomnia and depression and here our results are consistent [9,10,11,13]. However, population studies also show in unselected populations that OSA symptoms (snoring, sleepiness) are similar in men and women [8]. Our findings and those from sleep clinic samples of patients referred for investigation report significantly higher mean BMI in women compared to men [25,26]. We can speculate that the cause of the relative under-diagnosis of OSA in women in our sample is that men with symptoms of OSA or with a CVD history are more likely to be referred for investigation for a condition that is regarded as male-predominant. For women to be referred, the clinical picture may need to be overtly related to OSA. Further work is needed to determine the prevalence and clinical characteristics of OSA in women.

It is difficult to make prevalence comparisons with national data as the prevalence of OSA in Australian women in unclear. A 2013 study of 412 community-dwelling Australian women estimated a similar prevalence of moderate to severe OSA (apnea hypopnea index > 15/h) of 6%. However, this study used a single-channel sleep monitor that underestimates OSA severity [27], may underestimate prevalence, and does not match current standards for assessing OSA [28]. In the Busselton health study from 1990, of 102 women, 5 (5%) recorded a respiratory disturbance index ≥ 15/h. Given the increasing burden of obesity that has occurred since this study, these OSA estimates are likely underestimated.

Regardless, there is also a health burden in those without a diagnosis of OSA but who report OSA symptoms. In both genders, those with loud snoring and daytime sleepiness frequently reported witnessed apneas and also showed independent associations with depression, insomnia, and fair to poor general health. In men, OSA symptoms were also significantly associated with hypertension and nocturia. The prevalence of symptoms increases the potential OSA population over that diagnosed by 100% in men and 260% in women. Participants identified with OSA symptoms without a diagnosis experience very poor health status as their impairments in physical and mental health quality of life scores approximate those seen in arthritis and heart failure populations [29]. Our data suggests that sleep should be included when managing health-related lifestyle risks (smoking, exercise, hypertension), and that investigations for sleep apnea also need to be considered. Our findings support those of Lindberg et al., who have recently reported that women with OSA symptoms are less likely than men to be diagnosed and treated for OSA [7]. We therefore propose that a higher index of clinical suspicion of sleep apnea is required particularly in women who may not be inclined to report snoring and daytime sleepiness but who have other potential sleep symptoms.

A strength of this study is the sample that was derived from a random sample and that weighting of that sample may overcome the non-response in this follow-up assessment of the cohort [24]. Respiratory disease was based on self-report combined with spirometric measures obtained in a previous wave of data collection. Self-report of chronic conditions is a limitation of this study; however, misclassification of participants as “no condition” results in our findings being biased towards the null. Self-reported height is likely to be overestimated in older adults [30] leading to underestimates in BMI. Diabetes and hypertension were ascertained through self-report and medication use, which reduces recall bias, and self-reports of macrovascular events have been shown to be valid [31,32]. Notwithstanding effects of lifestyle changes, 77% of people self-reporting diabetes and 78% reporting hypertension or their treatment were determined at the previous clinic stage in 2008–2010 to have these disorders. We did not assess excessive daytime sleepiness (EDS) with the Epworth Sleepiness Scale. However, we have previously shown that objectively assessed OSA is not associated with Epworth scores but rather with a more broad description of sleepiness (at least two or three problems) as used in this study [33].

Menopause status and hormone replacement therapy (HRT) may influence the relationship of OSA with comorbidities. We did not assess menopause status, which prevents us from directly examining any relationship of menopause and OSA [34]. However, of note, 85% of women with diagnosed OSA were at least 55 years, and very likely to be post-menopausal, compared to only 41% of women in the OSA symptoms group. This suggests that pre-menopausal women may also be a target group for investigation for OSA in contrast to the commonly held assumption that OSA is a post-menopausal concern. HRT was used in only 5% of women in our study, of whom 60% were aged under 60 years, which is the age group showing a more favorable HRT-related risk profile for CVD and mortality [35,36]. Furthermore, given that over 70% of HRT use occurred in women with no diagnosis of OSA/OSA symptoms regardless of age group, HRT is unlikely to have been responsible for residual confounding in our analyses adjusted for age.

## 5. Conclusions

In conclusion, women were disproportionately less likely than men to receive a diagnosis of OSA based on the prevalence of possible undiagnosed OSA/symptoms in the population. When women were diagnosed, overt OSA characteristics were present suggesting different diagnostic processes for a condition generally regarded as affecting men. Nevertheless, there is a sizable population of men and women with possible undiagnosed OSA in the community who have major health impacts and warrant investigation. International studies in populations with relatively low prevalence of obesity (mean BMI ~ 25 kg/m^2^) suggests that moderate to severe OSA occurs in approximately 20% of women [1,2]. There are major challenges to better clinical care and cost-effective policy to address OSA in Australia. This first is to objectively determine the prevalence of OSA in women in Australia where nearly 30% of women are obese [37]. The second challenge is to correctly identify from within this potentially large community pool of OSA which women are at risk of OSA-related sequelae and therefore require treatment with a possible focus on presentation of insomnia symptoms. Women with symptoms of sleep problems are at risk of accidents, reduced work productivity and social functioning as well as the reductions in quality of life we have identified. A societal focus on identifying and ameliorating the drivers of poor quality sleep is appropriate given the huge direct and indirect costs of sleep problems to the community [38]. In addition, limited system resources will require novel screening strategies to identify, prevent, and reduce OSA-related disparities in health in Australian women.

## Figures and Tables

**Table 1:** Sleep, biomedical, and demographic characteristics of The North West Adelaide Health Study (NWAHS) participants (% (n)) by sex and overall.

	Male (n = 753)	Female (n = 808)	*p*	Total
**Sleep disorders and symptoms, % (n)**				
Diagnosed OSA	12.6 (95)	3.3 (27)	<0.001	7.8 (122)
Snoring: loud or bothered others	38.4 (285)	20.6 (161)	<0.001	29.3 (446)
Witnessed breathing pauses	15.9 (118)	10.0 (78)	0.002	12.6 (196)
≥2 of 3 daytime sleepiness problems	37.9 (286)	38.7 (312)	0.77	38.3 (598)
Loud snoring and ≥2 daytime sleepiness problems	17.1 (129)	9.7 (78)	<0.001	13.3 (207)
Insomnia Severity Index ≥10	16.2 (120)	21.3 (165)	0.010	18.8 (285)
Nocturia	28.6 (212)	32.4 (253)	0.11	30.6 (465)
Body mass index (kg/m^2^)			<0.001	
≤24.9	23.8 (179)	36.1 (283)		30.1 (462)
25.0–29.9	47.3 (356)	32.5 (255)		39.8 (611)
≥30.0	28.9 (217)	31.5 (247)		30.2 (464)
Current smoker	13.0 (98)	10.8 (87)	0.27	11.9 (185)
**Demographics, % (n)**				
Age (mean (SD))	53.2 (14.8)	54.9 (15.5)	0.027	54.1 (15.2)
Highest educational attainment			<0.001	
High school or less	34.8 (262)	46.2 (373)		40.7 (635)
Certificate (including Trade) or Diploma	38.9 (293)	23.9 (193)		31.1 (486)
Bachelor degree or higher	25.0 (188)	26.0 (210)		25.5 (398)
Not stated	1.3 (10)	4.0 (32)		2.7 (42)
Annual household income			<0.001	
<$40,000	19.5 (147)	27.6 (223)		23.7 (370)
$40,001–$80,000	24.6 (185)	21.8 (176)		23.1 (361)
≥$80,001	47.7 (359)	34.0 (275)		40.6 (633)
Not stated	8.2 (62)	16.6 (134)		12.6 (196)
Financial stress ^1^			0.18	
Yes	74.0 (557)	73.1 (591)		73.5 (1148)
No	19.8 (149)	16.2 (131)		17.9 (280)
Not stated	6.2 (47)	10.6 (86)		8.5 (133)
Partner/relationship	77.2 (581)	66.2 (535)	<0.001	71.5 (1116)
Private health insurance	61.9 (466)	66.2 (488)	0.134	61.1 (954)

^1^ Financial stress (in relation to family’s money situation): Yes: spends more money than is earned; have just enough money to get through to the next pay day/some money left over each week but it is spent/saves a bit every now and then; No: can save a lot. OSA = obstructive sleep apnea.

**Table 2:** Symptom and demographic characteristics (% (n)) of participants with diagnosed obstructive sleep apnea (OSA) and OSA symptoms (snoring and daytime sleepiness ^1^) not previously diagnosed, by sex.

	Males			Females		
	No OSA	Diagnosed OSA	OSA Symptoms not Diagnosed	No OSA	Diagnosed OSA	OSA Symptoms not Diagnosed
	(n = 563)	(n = 95)	(n = 96)	(n = 709)	(n = 27)	(n = 71)
Snoring: loud/bothered others	23.4 (129)	63.8 (60) ^3^	100.0 (96)	11.0 (75)	59.3 (16) ^3^	100.0 (71)
Witnessed breathing pauses	4.0 (22)	66.7 (62) ^3^	35.1 (34) ^3^	6.9 (47)	70.4 (19) ^3^	18.3 (13) ^3^
≥2 of 3 sleepiness problems ^1^	25.6 (144)	48.4 (46) ^3^	100.0 (96)	32.1 (228)	48.1 (13)	100.0 (71)
Loud snoring and ≥2 sleepiness problems	0.0 (0)	35.8 (34)	100.0 (96)	0.0 (0)	25.9 (7)	100.0 (71)
Insomnia Severity Index ≥10	13.7 (77)	18.9 (18)	27.1 (26) ^3^	17.3 (123)	29.6 (8)	52.1 (37) ^3^
Nocturia	24.0 (132)	41.5 (39) ^3^	43.2 (41) ^3^	30.9 (211)	25.9 (7)	49.3 (35) ^2^
BMI (kg/m^2^) ≥30.0	23.6 (133)	52.6 (50) ^3^	35.8 (34)	27.8 (191)	77.8 (21) ^3^	50.7 (36) ^3^
BMI (mean (SD))	27.6 (4.3)	30.8 (5.5) ^3^	29.5 (4.3) ^3^	27.5 (6.0)	34.4 (6.5) ^3^	30.1 (7.6) ^2^
Neck circumference >43 cm	28.3 (120)	41.6 (32) ^3^	29.3 (32) ^3^	4.0 (20)	31.8 (7) ^3^	12.2 (6) ^3^
Current smoker	10.2 (57)	17.0 (16)	27.1 (26) ^3^	11.2 (78)	18.5 (5)	7.0 (5)
Insufficient physical activity	29.9 (159)	37.8 (39)	50.0 (45)	41.5 (275)	75.0 (18) ^3^	65.1 (41) ^2^
Hormone replacement therapy				4.4 (31)	7.1 (2)	12.7 (9) ^3^
Age (mean (SD), years)	52.2 (15.1)	62.0 (10.9)	50.4 (14.0)	54.8 (15.8)	63.3 (10.7) ^2^	52.6 (11.9)
Age ≥55 years	37.1 (209)	71.3 (67) ^3^	35.7 (34)	44.1 (313)	85.2 (23) ^3^	40.8 (29)
Married/partner	74.5 (420)	76.6 (72)	93.7(89)	65.4 (464)	66.7 (18)	73.2 (52)
Private health insurance	63.1 (355)	51.6 (49) ^2^	64.2 (62)	61.5 (436)	42.9 (12)	56.3 (40)

Abbreviations: BMI = body mass index, ^1^ at least two of the following three problems: Feels tired, fatigued, sleepy; Feels sleepy if sitting quietly during day/early evening; Trouble staying awake while driving, eating, social activities. ^2^ *p* < 0.05 compared to no OSA. ^3^ *p* < 0.01 compared to no OSA.

**Table 3:** Prevalence of chronic conditions (%, (n)) in participants with diagnosed of OSA or with OSA symptoms (snoring and daytime sleepiness ^1^) not previously diagnosed, by sex.

Chronic Condition, % (n)	No OSA	Diagnosed OSA	OSA Symptoms Not Diagnosed	Total, %
**Male**	(n = 563)	(n = 94)	(n = 95)	
*Doctor*-*Diagnosed*				
Diabetes (or medication)	8.7 (49)	26.6 (25) ^3^	9.5 (9)	11.0
Cardiovascular disease/stroke	6.9 (39)	21.3 (20) ^3^	8.3 (8)	8.9
Hypertension (or medication)	27.5 (155)	23.9 (20) ^3^	40.6 (39) ^3^	32.8
High cholesterol	23.4 (132)	36.2 (34) ^3^	33.3 (32) ^2^	26.3
Kidney disease	1.8 (10)	10.5 (10) ^3^	1.0 (1)	2.8
Obstructive lung disease	25.4 (143)	27.7 (26)	16.7 (16)	24.6
Depression (CESD)	20.7 (114)	23.9 (22)	34.4 (33) ^3^	22.9
SF-1 fair-poor general health	10.8 (60)	30.1 (28) ^3^	37.9 (36) ^3^	16.7
**Female**	(n = 710)	(n = 27)	(n = 71)	
*Doctor*-*Diagnosed*				
Diabetes (or medication)	8.0 (57)	22.2 (6) ^2^	5.6 (4)	8.3
Cardiovascular disease/stroke	5.6 (40)	14.8 (4)	5.6 (4)	5.9
Hypertension (or medication)	33.1 (235)	48.1 (13)	26.8 (19)	33.1
High cholesterol	28.5 (202)	63.0 (17) ^3^	32.4 (23)	30.0
Kidney disease	1.8 (13)	0.0 (0)	4.2 (3)	2.0
Obstructive lung disease	29.4 (209)	48.1 (13) ^2^	21.1 (15)	29.3
Depression (CESD)	29.0 (196)	33.3 (9)	64.3 (31) ^3^	32.3
SF-1 fair-poor general health	18.8 (132)	46.2 (12) ^3^	47.8 (33) ^3^	22.2

Abbreviations: CESD = Center for Epidemiologic Studies Depression Scale ≥16; SF-1: Question one of the SF-36: In general, would you say your health is? (Excellent; very good; good; fair; poor). ^1^ at least two of the following three problems: Feels tired, fatigued, sleepy; Feels sleepy if sitting quietly during day/early evening; Trouble staying awake while driving, eating, social activities. ^2^ *p* < 0.05 compared with no OSA by Pearson Chi Square test. ^3^ *p* < 0.01 compared to no OSA by Pearson Chi Square test.

**Table 4:** Unadjusted mean (SD) physical component summary (PCS) and mental component summary (MCS) scores and age- and BMI-adjusted coefficients for the association of OSA status with PCS and MCS scores.

	No OSA	Diagnosed OSA	OSA Symptoms Not Diagnosed	Total
Unadjusted mean scores				
Males				
PCS ^1^	49.6 (8.9)	44.8 (11.4) ^2^	43.7 (11.5) ^2^	48.2 (9.9)
MCS ^1^	49.6 (9.4)	46.2 (11.4) ^2^	45.7 (10.2) ^2^	48.7 (9.9)
Females				
PCS ^1^	48.2 (9.9)	37.9 (12.2) ^2^	40.5 (9.8) ^2^	47.2 (10.3)
MCS ^1^	47.0 (10.2)	40.9 (11.6) ^2^	36.4 (11.9) ^2^	45.9 (10.9)
Linear regression Unstandardized B coefficients				
Males				
PCS		−1.2 (−3.1, 0.6)	−4.0 (−6.3, −1.6) ^3^	
MCS		−1.8 (−3.8, 0.21)	−5.3 (−7.7, −2.8) ^3^	
Females				
PCS		−4.6 (−7.8, −1.3) ^3^	−6.4 (−9.1, −3.8) ^3^	
MCS		−3.5 (−7.0, 0.03)	−8.1 (−11.0, −5.2) ^3^	

^1^ ANOVA or Welch *p* ≤ 0.001 across group means; ^2^ *p* < 0.05 compared to no OSA; ^3^ unstandardized coefficient *p* < 0.01 representing reduction in PCS and MCS scores for diagnosed OSA or OSA symptoms compared to those categorized as No OSA.
